# Optical characterization of 3D printed PLA and ABS filaments for diffuse optics applications

**DOI:** 10.1371/journal.pone.0253181

**Published:** 2021-06-16

**Authors:** Caterina Amendola, Michele Lacerenza, Ileana Pirovano, Davide Contini, Lorenzo Spinelli, Rinaldo Cubeddu, Alessandro Torricelli, Rebecca Re

**Affiliations:** 1 Dipartimento di Fisica, Politecnico di Milano, Milan, Italy; 2 Istituto di Fotonica e Nanotecnologie (IFN), Consiglio Nazionale delle Ricerche, Milan, Italy; Ningbo University, CHINA

## Abstract

The interest for Fused Deposition Modelling (FDM) in the field of Diffuse Optics (DO) is rapidly increasing. The most widespread FDM materials are polylactic acid (PLA) and acrylonitrile butadiene styrene (ABS), thanks to their low cost and easiness-to-print. This is why, in this study, 3D printed samples of PLA and ABS materials were optically characterized in the range from the UV up to the IR wavelengths, in order to test their possible employment for probe construction in DO applications. To this purpose, measurements with Near Infrared Spectroscopy and Diffuse Correlation Spectroscopy techniques were considered. The results obtained show how the material employed for probe construction can negatively affect the quality of DO measurements.

## Introduction

Diffuse Optics (DO), is a branch of optics, originally developed in the atmosphere and biomedical fields, which makes use of light in the VIS and NIR spectral range to optically characterize, in terms of absorption and scattering coefficients, turbid media such us: human tissues (e.g. brain for non-invasive assessment of the hemodynamic cortical activation or the oxidative metabolism status) [1], fruits (e.g. for non-destructive maturity assessment) [2], pharmaceutical tablets and wood (e.g. for quality control during production) [3]. In DO measurements light is typically delivered to the sample by optical fiber (injection fiber) and diffusely remitted photons are collected by another optical fiber (collection fiber) set at a relative distance of few centimetres. Injection and collection fibers are usually hosted in a probe, a housing which must guarantee good adhesion with the sample, and ambient light shielding.

With the advent of the Fused Deposition Modelling (FDM) technology, the use of a 3D filament printer for building a custom probe for each DO instrument and application is significantly increased [4–8]. In the wide context of the additive manufacturing sector, the FDM, is the most spread 3D printing methodology, because of the low printer and filament costs and the usage simplicity compared to other 3D printer techniques such as stereolithography (SLA) or selective laser sintering (SLS) [9]. In FDM a thermoplastic polymer, in the form of a filament, is extruded on a plate adding layer by layer slices of fused filament. The geometry and structure of the 3D object created are defined by sliced model (g-code) previously defined by a software. The printer settings are strongly dependent on the kind of the filament employed, and the choice of the filament’s material depends, in turn, on the final application of the printed object [10]. The two materials, which are mostly used in FDM, are polylactic acid (PLA) and acrylonitrile butadiene styrene (ABS), due to their great trade-off between cost and easiness-to-print.

An optical characterization of the materials used in FDM is necessary to understand the actual possibility of their employment in this contest. With this work, we would like to better investigate the optical spectral characteristics of PLA and ABS materials, which were widely characterized from a mechanical and thermal point of view, but not from an optical one. In particular, there is a lack of knowledge on the optical characteristics of the final printed object properties. We will also assess the applicability of 3D printed materials in creating tools for DO applications such as probes, phantoms’ boxes or components for detectors supports.

In the next sections we will list the materials we have chosen to test. These were 3D printed in the form of thin sheets (about 0.3–0.5 mm) and we will present the optical spectra of these samples obtained with a spectrophotometer in the range 400–1300 nm. We also tested the use of a couple of PLA filaments for practical applications. For this purpose, we created a probe for a Time-Domain (TD) near infrared spectroscopy (NIRS) [11] device and a diffuse correlation spectroscopy (DCS) [12] instrument, two techniques widely employed in the DO field. We evaluated the effect of the transmitted radiation, if any, on the measurements on a synthetic phantom, to mimic its effect on a real measurement.

## Materials and methods

### 3D printed samples

PLA and ABS sheets (parallelepiped of 0.3 mm thickness, and 20x20 mm side) were designed with Inventor Pro 2019 (Autodesk Inc., US) software. They were printed with a 3D commercial filament printer (Sharebot NG, Nibionno (LC), Italy; Filament diameter 1.75 mm). The sheets were printed as two layers with 100% rectilinear infill. The nozzle diameter was 0.35 mm and the extrusion multiplier was set to 0.9. During the printing of the first layer the cooling fan acting on the deposited material was off, and it was at its maximum power for the rest of the time. For the PLA filaments the extruder temperature was set to 205°C and printing speed to 3800 mm/min, whereas for ABS filament the extruder temperature was raised to 250°C, the bed was heated up to 80°C, and printing speed was reduced to 2800 mm/min. To avoid any contamination of the printed objects, no adhesive material or spray have been used to facilitate its attachment to the printing plate, but a 3D printing surface (Lokbuild, Coalville, UK) was inserted on the plate. The printed sample sheets have been always handled with gloves. In Table 1, the details of the different filaments we tested are presented. In particular, we reported the colors and the producers of the filaments, together with the thicknesses of the printed sheets, as measured with a screw micrometer. The white filament from MDKOEM was also printed at different extruder’s temperatures: 185°C, 190°C, 195°C, 200°C, 210°C and 215°C. The obtained sheets’ thicknesses were: 0.34 mm, 0.33 mm, 0.35 mm, 0.34 mm, 0.31 mm and 0.33 mm, respectively.

**Table 1:** Different kind of filaments employed for printing the sheets.

Filaments Type					
PLA			ABS		
Colour	Thickness [mm]	Producer	Colour	Thickness [mm]	Producer
Black	0.35	FILOALFA	Black	0.30	NPS S.r.l.
Black	0.36	3DiITALY	Black	0.31	3DiTALY
Black	0.52	MKOEM	White	0.25	FILOALFA
White	0.35	FILOALFA	White	0.33	NPS S.r.l.
White	0.43	MDKOEM	Blue	0.33	NPS S.r.l.
Neutral	0.35	FILOALFA	Light gray	0.23	FILOALFA
Light Blue	0.49	MDKOEM	Red	0.35	Filoprint
Transparent blue	0.53	MDKOEM			
Blue	0.42	MDKOEM			
Grey	0.47	MDKOEM			
Metal Grey	0.39	MDKOEM			
Light Grey	0.30	3DACTIVE			
Yellow	0.53	MDKOEM			
Red	0.52	MDKOEM			
Red	0.43	TIANSE			
Green	0.51	MDKOEM			
Pink Transparent	0.58	MDKOEM			
Transparent	0.40	MDKOEM			

### Optical characterization

A UV/VIS/NIR spectrophotometer (V570, Jasco Inc., Easton, USA) was employed for obtaining the spectrum of the different materials in the wavelength range 400–1300 nm. The spectrophotometer settings were: bandwidth = 1 nm (NIRS = 4 nm); data pitch = 2 nm; light source changeover = 340 nm; detector changeover = 880 nm; all the spectra were collected at room temperature in air.

The spectra measurements were obtained as percentage transmittance (T_%_). According to the modified Lambert Beer law [13], we can express T_%_ in terms of both the absorption (μ_a_) and scattering coefficient (μ_s_) of the material considering an extinction coefficient μ = μ_a_+μ_s_ [14] expressed as: μ = 1/d∙*log*_10_(100/*T*_%_)∙ln(10) here d is the actual thickness of the sheet.

### 3D printed probe for diffuse optics

In order to verify the impact of the different materials tested in DO applications, we choose two PLA black filaments from two different producers: FILOALFA and 3DiTALY. The instruments employed for the measurements and the characteristic of the probes printed for the TD-NIRS and DCS devices are described below.

**Fig 1:**
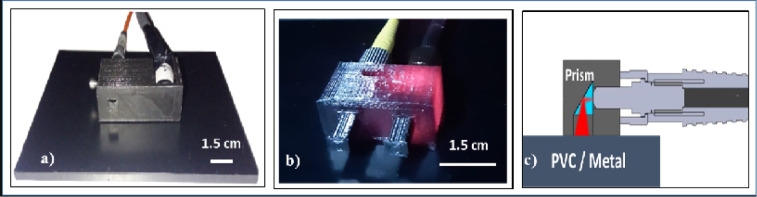
Probe for optical fibers. a) TD-NIRS probe; b) DCS probe (FILOALFA); c) probe’s schematic.

NIRS measurements were performed with a TD device previously developed at the Department of Physics, Politecnico di Milano [15]. It employs two wavelengths at 689 nm and 828 nm with an average output power of 3 mW (8.5 mW) @689 nm (828 nm). TD-NIRS probe, shown in Fig 1A), was printed as described in [16] (20% rectilinear infill, 2 top and bottom layers) with just one interfiber distance for simplicity (ρ = 30 mm). For the injection channel a BK7 glass right angle prism, with 5 mm x 5 mm silver coated reflecting surface (Lambda research optics. Inc., California, USA), was inserted in order to bend the incident light onto the sample of 90°.

The DCS instrument was developed at the Department of Physics, Politecnico di Milano, combining a commercial highly coherent continuous wave laser, operating at 784 nm with 120 mW of maximum power, and coherence length higher than 8 m (Toptica photonics, Germany). The detection chain is comprehensive of a single photon avalanche diode detector (Excelitas technology, Canada) and a digital correlator (ALV, Germany). The probe for DCS measurements, shown in Fig 1B) was printed with the same characteristics of the NIRS probe, but with an interfiber distance of 15 mm and two prisms: one for bending the injection light and one for the detected one. The schematic of the prism’s housing is shown in Fig 1C).

We performed two set of measurements to evaluate the detected signal in terms of counts/s with both the techniques and the two PLA filaments: 1) over a reflecting metallic surface and 2) over a totally black absorbing Polyvinyl chloride (PVC) surface (Fig 1A). In both cases we performed 30 repeated measurements with an acquisition rate of 1 s. We qualitatively compared the signals measured on the two surfaces, to evaluate the possible presence of stray light propagating in the probe itself, which could contaminate the performed measurements.

We also tried to understand the effect of the stray light propagating in the probe on the final outcomes of the TD-NIRS and DCS measurements, *i*.*e*. on the optical properties and distribution time of flight (DTOF) and the autocorrelation function, respectively. For TD-NIRS we performed 30 repeated measurements, on a solid calibrated homogeneous phantom (nominal values of μ_a_ = 0.14 cm^-1^ and reduced scattering coefficient μʹ_s_ = 20 cm^- 1^, @660 nm), with both the printed probes. The injected power was set in order to have 10^6^ counts/s for each acquisition. The experimental DTOFs were fitted with an analytical solution of the diffusion equation for a semi-infinite homogeneous medium to estimate μ_a_ and μʹ_s_ [14].

For the DCS measurements, a liquid phantom (μ_a_ = 0.11 cm^-1^, μʹ_s_ = 10.6 cm^-1^, diffusion coefficient D_b_ = 1.2 10^−8^ cm^2^/s, @ 785 nm) was prepared. As in the previous case, 30 measurements of 1 s were acquired employing each probe. The electric field autocorrelation function was analyzed with a semi-infinite homogeneous model for photon migration [1, 17] and the diffusion coefficient D_b_ was estimated. The optical properties retrieved with the TD-NIRS instrument [8], were used in the fitting procedure for determining D_b_.

## Results

### Optical characterization

**Fig 2:**
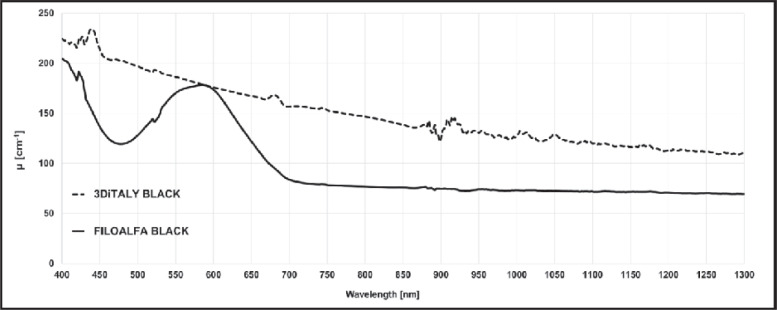
Extinction coefficient *μ* spectra in the wavelength range 400–1300 nm for two black PLA filaments (3DiTALY, dashed line; FILOALFA solid line).

In this section, the extinction coefficient spectra of the different sheets are presented. The spectra are organized in different graphs to better highlight several key points of the results. In Fig 2, the spectra for two black PLA samples, printed with filaments of two different producers (3DiTALY and FILOALFA), are presented. The extinction coefficient is much higher for the 3DiTALY PLA sample in all the wavelength range. These two filaments were chosen for printing the probes described in the previous section to appreciate the effects of their different spectra behaviour in DO acquisitions (see next section).

**Fig 3:**
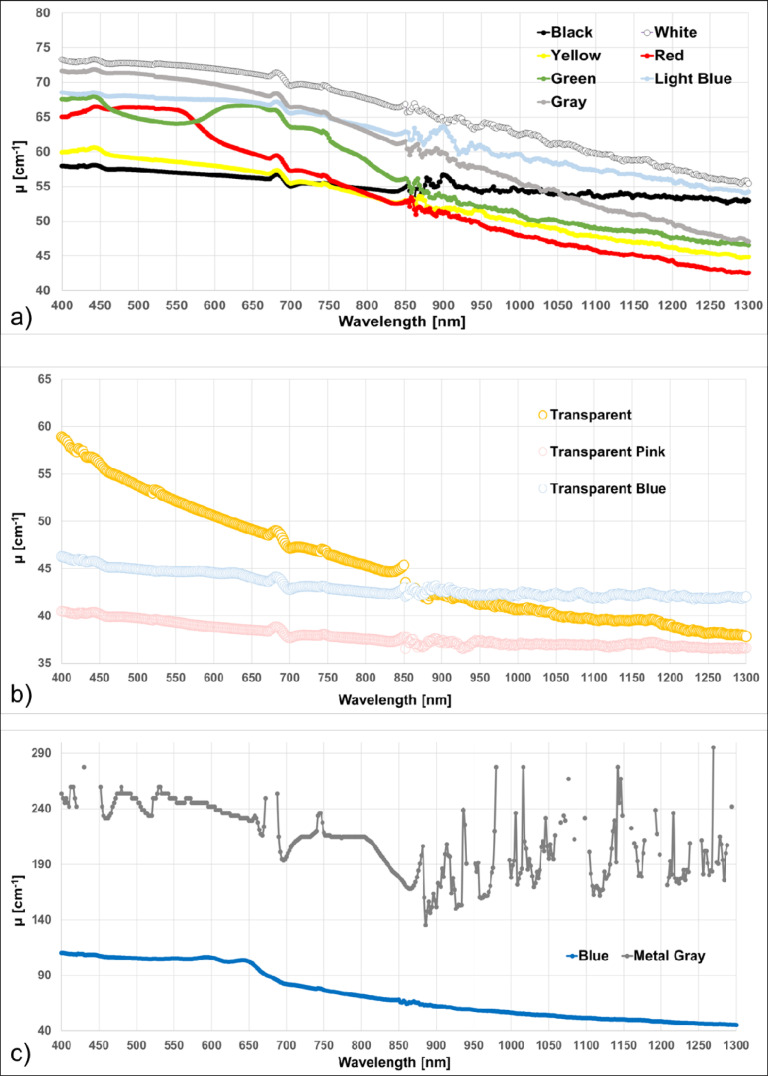
Extinction coefficient *μ* spectra in the wavelength range 400–1300 nm for PLA filament with different colors from the same producer (MDKOEM). a) opaque colors; b) transparent colors; c) metal gray and blue.

In Fig 3, μ for PLA samples with different colours but from the same producer (MDKOEM) are represented. For a better visualization in Fig 3A μ for the opaque colours is shown, in Fig 3B for transparent ones, while in Fig 3C for the metal gray and the blue filaments.

**Fig 4:**
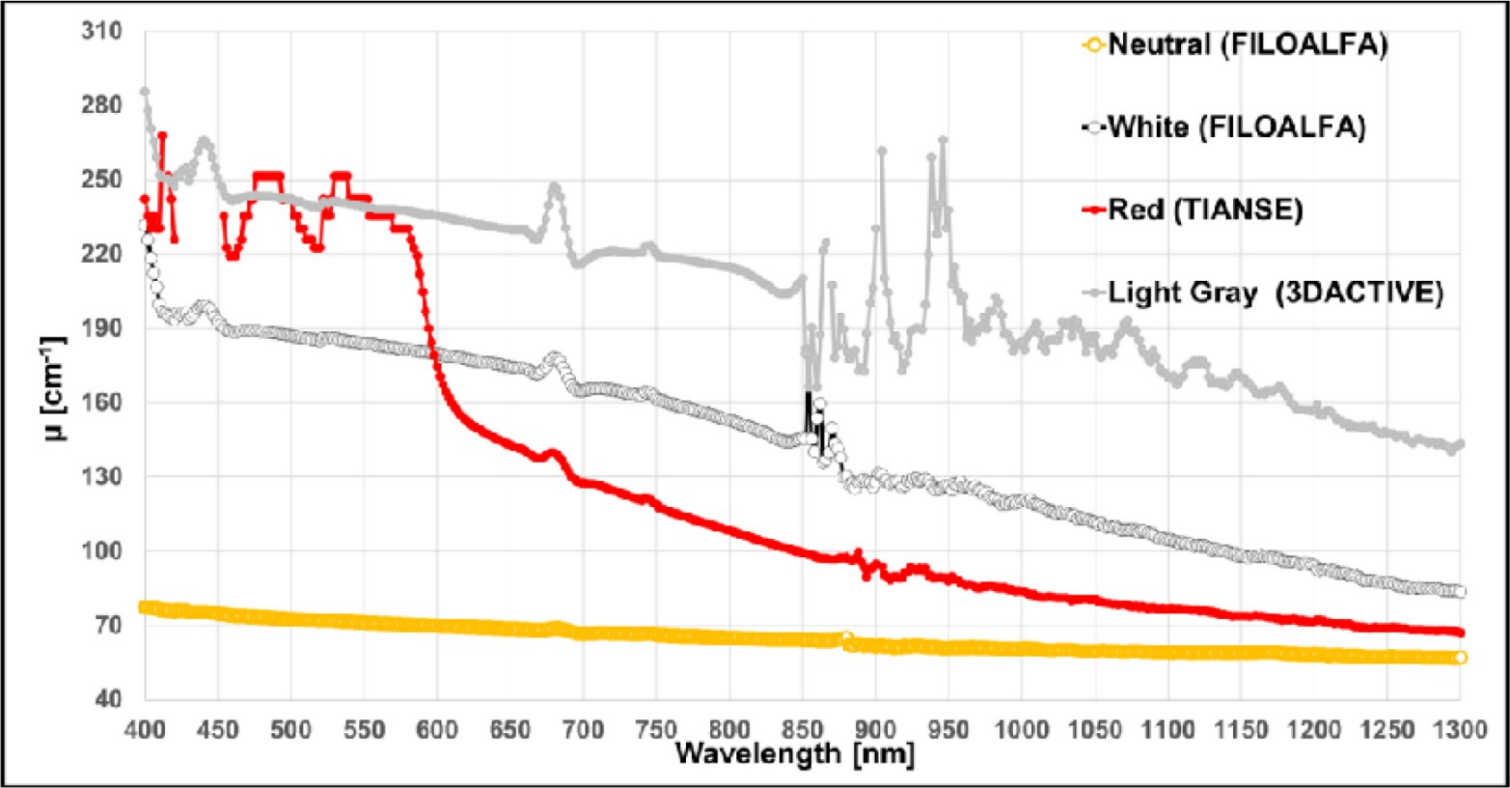
Extinction coefficient *μ* spectra in the wavelength range 400–1300 nm for PLA filaments with different colors from diverse producers.

The μ of the remaining PLA samples are shown in Fig 4. In the previous graphs, the signal at some wavelengths (in the range between 430 and 450nm, 650 and 700 nm and >900 nm) was extremely low, making these points not reliable (points removed from the graphs).

**Fig 5:**
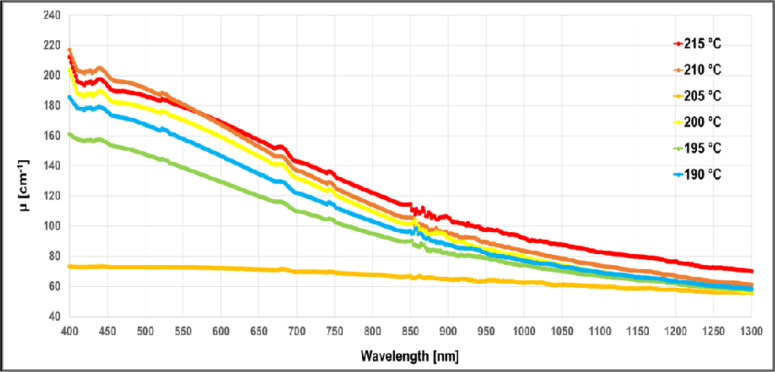
Extinction coefficient *μ* spectra in the wavelength range 400–1300 nm for the white PLA filament from MDKOEM, printed at different extruder’s temperatures.

In Fig 5 the behaviour, in terms of μ, of samples printed at different extruder temperature with the same filament (white PLA from MDKOEM), is shown. We verify that for a temperature lower than 190°C the sheet was not well printed, for this reason the curve at 185°C is not shown.

**Fig 6:**
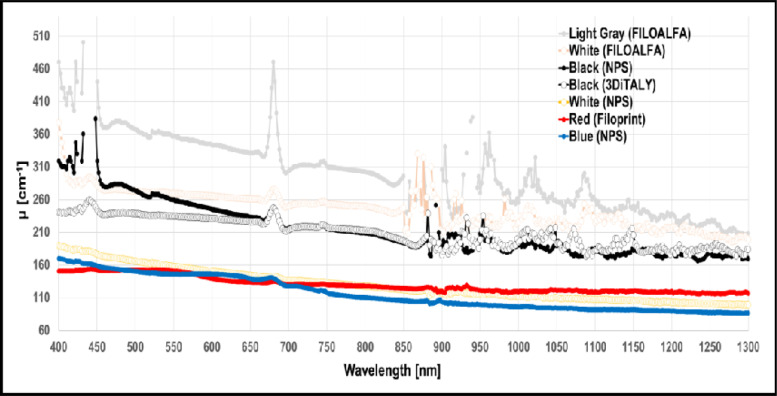
Extinction coefficient *μ* spectra in the wavelength range 400–1300 nm for ABS filaments with different colors from diverse producers.

Finally, in Fig 6 the μ for the ABS samples of different colours and from different producers are represented.

### Application in diffuse optics: TD-NIRS and DCS probes characterization

**Fig 7:**
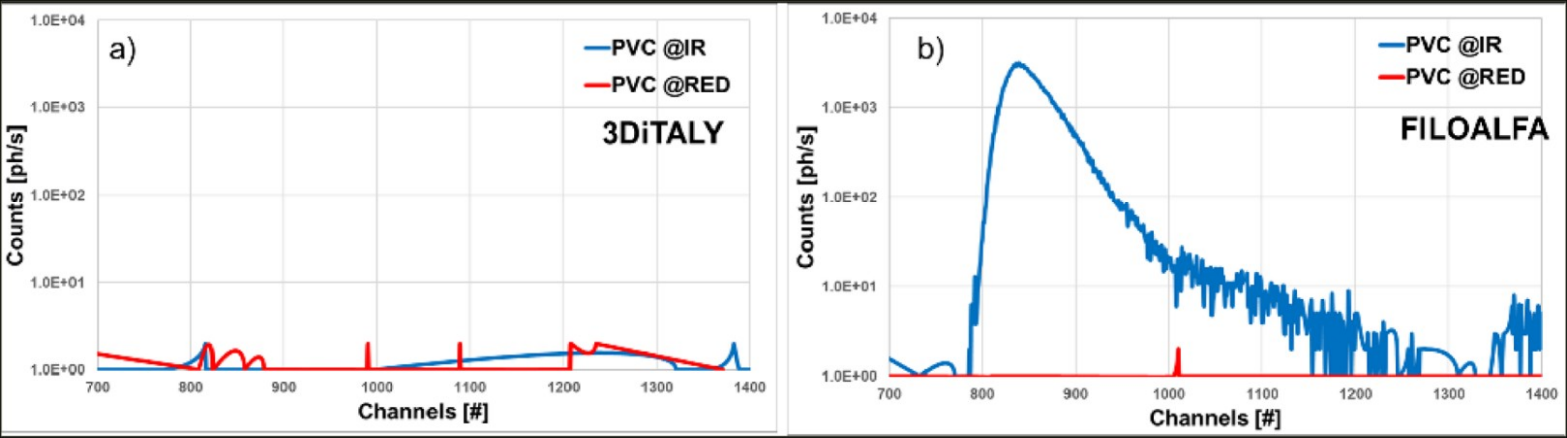
TD-NIRS signals obtained with the two 3D printed probes tested on the PVC surface at 689 nm (RED) and 828 nm (IR). In panel a) results are shown for probe printed with black 3DiTALY filament; in panel b) for probe printed with black FILOALFA filament.

In Fig 7, we can observe the signals acquired with the TD-NIRS device with the two 3D printed probes, tested at two wavelengths on a PVC surface. In the figures we have represented one of the 30 curves acquired. For the probe printed with the black 3DiTALY PLA (Fig 7A), no signal could be observed, except for the detector background noise. All the light injected into the PVC surface was absorbed and no backscattered photons could be detected. On the contrary, the probe printed with the black FILOALFA filament gave origin to spurious signal at the infrared wavelength (Fig 7B). For what concern the acquisitions on a metallic surface (data not shown, but available on 10.5281/zenodo.4736126), not negligible signals were found both @689 nm and @828 nm.

**Fig 8:**
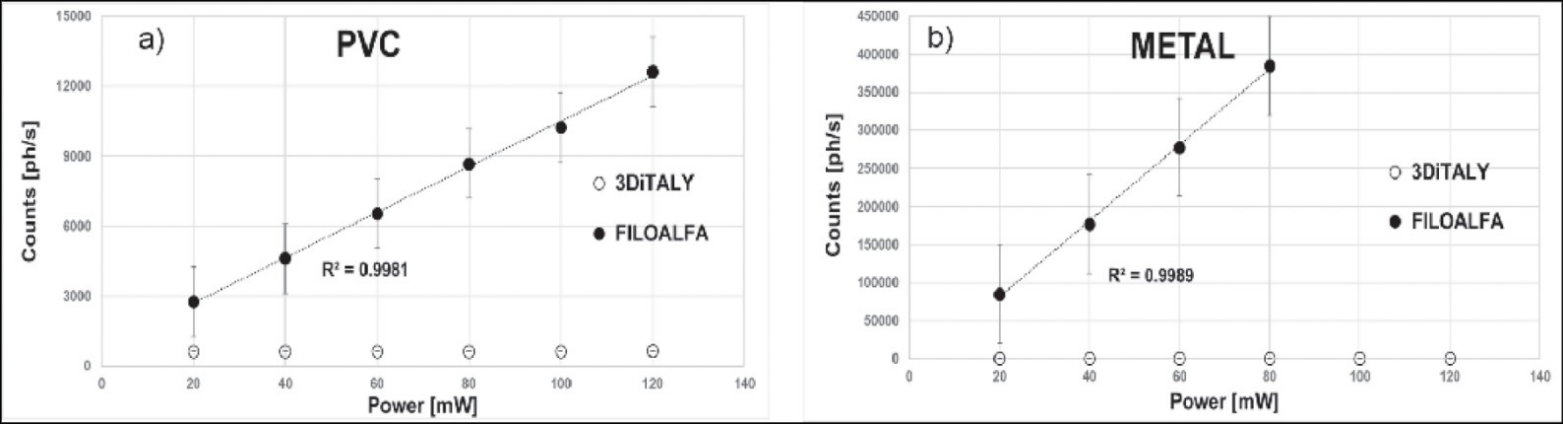
Photon counts obtained with the two 3D printed probes tested against a PVC (a) and METAL (b) surface. The dotted lines are the linear fit.

In Fig 8 the total number of counts (and the relative standard deviations) measured with the DCS device, placing the two probes on PVC (a) and metal (b) surfaces are presented, as function of the injected power. It can be noticed a significant signal amount only for the FILOALFA probe with both the surfaces. The detected signal increases linearly with the injected power (R^2^ = 0.99 of the linear fit), indicating that the 3D printed material is not properly shielding the light, which is diffused inside the probe and detected by the system. The total number of counts is higher when the probe is placed on the metallic surface and we stop the acquisition for the FILOALFA probe at 80 mW.

### Applications in diffuse optics: Phantom measurements with TD-NIRS and DCS

**Fig 9:**
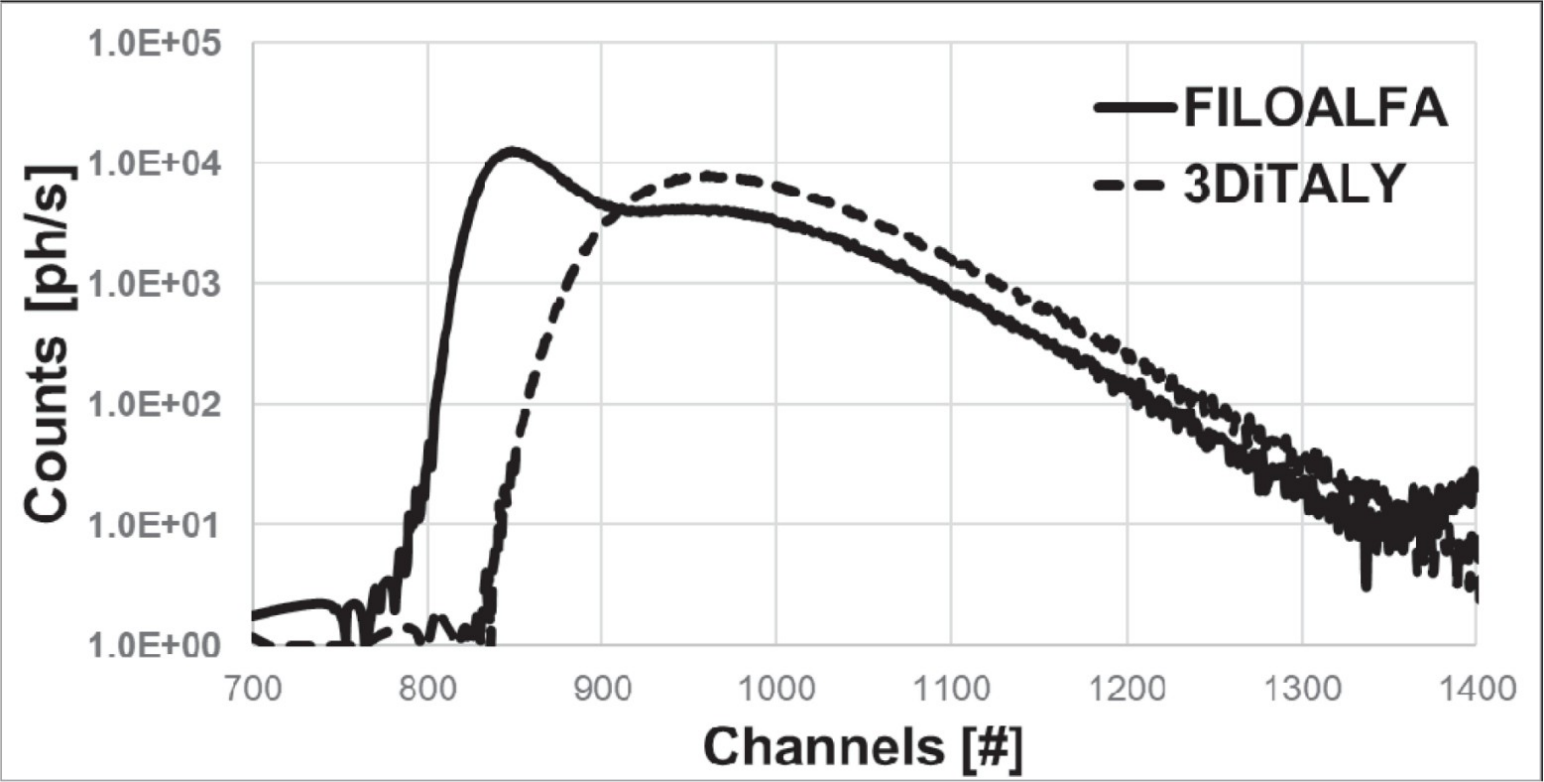
DTOFs acquired on the calibration phantom with two probes printed with different PLA filaments. Dashed line: 3DiTALY filament; solid line: FILOALFA filament. On the x-axis, the channels are the time bins of the time-correlated single-photon counting board used for the acquisitions: Each channel corresponds to 8 ps.

In Fig 9, we plotted a typical DTOFs acquired on the calibrated phantom at 828 nm with both probes. In the DTOF curve acquired with the FILOALFA probe (solid line), an unwanted peak is clearly visible.

In Table 2, the results of the TD-NIRS measurements on the calibrated solid phantom (nominal optical properties @660: μ_a_ = 0.14 cm^-1^, μʹ_s_ = 20 cm^-1^) are reported for the μ_a_, μʹ_s_ and χ^2^, for both the probes and the wavelengths. We can observe that the fitting procedure fails for the DTOFs acquired at 828 nm with the FILOALFA probe.

**Table 2:** Average absorption (μ_a_), reduced scattering (μ’_s_) coefficients and χ^2^ calculated over 30 repeated DTOFs acquired at 689 nm (RED) and 828 nm (IR) with two PLA probes.

	*μ*_*a*_ [cm^−1^]	*μ′*_*s*_ [cm^−1^]	χ^2^
3DiTALY @RED	0.11	13.34	1.48
FILOALFA @RED	0.11	14.54	1.30
3DiTALY @IR	0.10	10.84	1.21
FILOALFA @IR	0.05	1.02	2018.01

In addition, we repeated the tests on a phantom with lower μʹ_s_ (μ_a_ = 0.1 cm^-1^ and μʹ_s_ = 5 cm^-1^). We observed less influence in the shape of the curves, even at 828 nm, but the difference between the optical properties retrieved for the two materials was of 10%.

**Fig 10:**
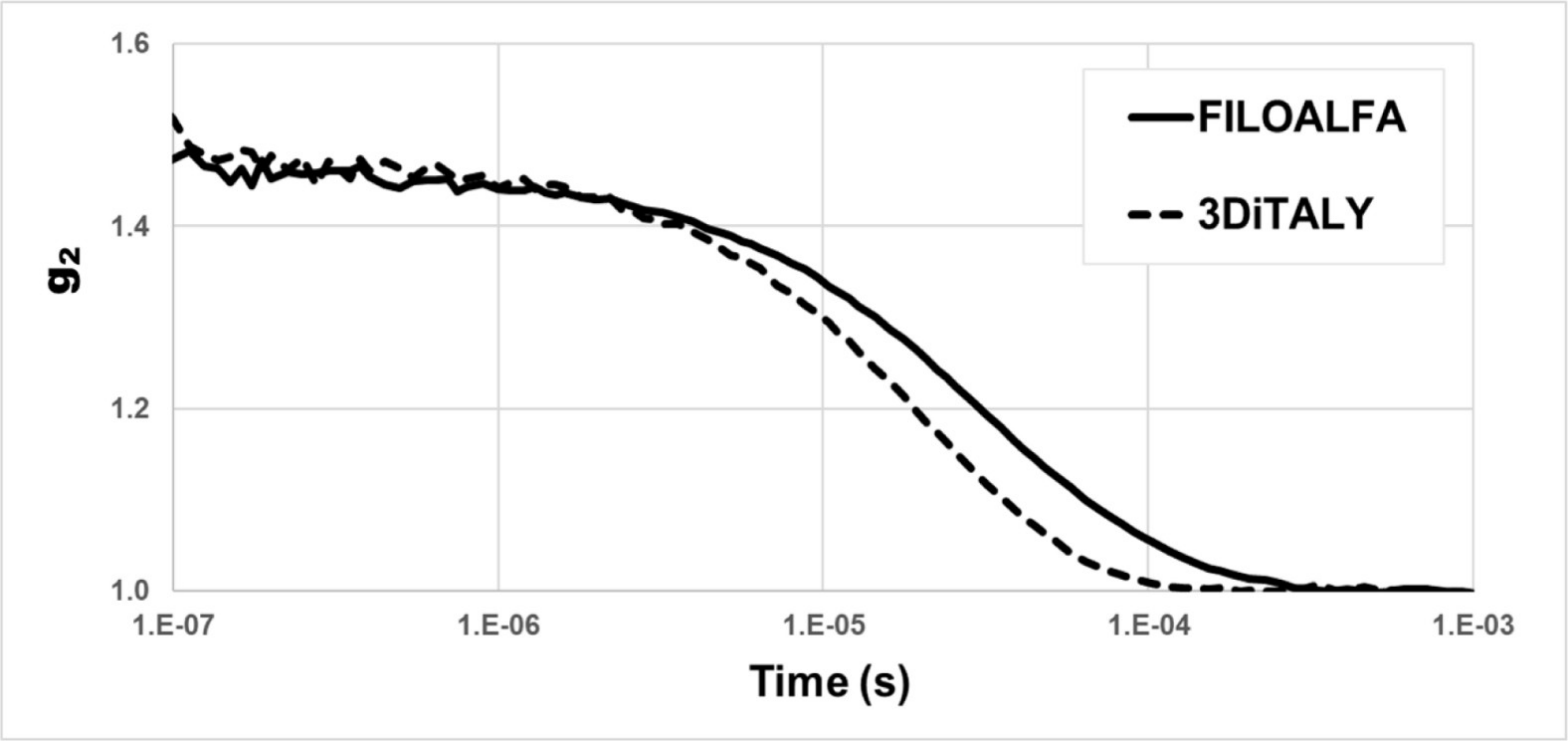
Intensity autocorrelation functions acquired with two probes printed with different PLA filaments: FILOALFA (solid line) and 3DiTALY (dashed line).

In Fig 10, the intensity autocorrelation curves, acquired with the DCS device placing both the probes on the liquid phantom, are shown. The decay time of autocorrelation function, generated when FILOALFA probe is used, is visibly higher than the one measured when 3DiITALY probe is employed. Furthermore, the estimated D_b_ was nearly double, and more similar to real values ((1.2±0.03)∙10^−8^ cm^2^/s) when measurements were acquired with 3DiTALY probe with respect to the FILOALFA one; in particular, we obtained D_b_ = (0.63±0.03)∙10^−8^ cm^2^/s for FILOALFA and D_b_ = (1.16±0.06)∙10^−8^ cm^2^/s for 3DiTALY.

## Discussion

PLA is one of the most spread filament materials for 3D printing thanks to the low printing process complexity, the moderate extrusion temperatures (180–220°C), and to the fact that it is not toxic. It is a degradable thermoplastic material, derived from biomass and approved for medical applications. This material was already widely characterized from a mechanical and thermal point of view but there is a lack of knowledge regarding its optical behaviour, in particular regarding the final 3D printed object. In literature, we can find some studies about the raw material. The behaviour of its index of refraction was studied from 300 to 1300 nm, observing a reduction from 1.499 to 1.440 [18]. Also the optical absorbance of the raw PLA has been investigated in the range 350–800 nm, and it was found always <0.12, so that it is possible to affirm that PLA is a transparent material [19]. Feng *et al*. measured 1 g of polymer powder and melted it in an oven at 130°C under vacuum for 12 h. The crucial point is that the heated powder is a different sample than the PLA printed with a 3D printer. In latter case, at first, the material must be extruded to create the filament, and then heated again to be printed on the plate: both these processes could change the sample optical properties, because PLA is susceptible to both hydrolytic and thermal degradation. Regarding the printed PLA, with a filament printer, they provide an X-ray diffraction (XRD) analysis, which is different from a spectroscopic one. They show that the printed polymers were amorphous, indicating that probably the cooling process did not lead to the formation of crystalline structures, which are relevant for the transparency of the printed sample. In Fig 2 we showed that two PLA printed samples using filaments of the same colour, but from different producers, have a completely different spectral behaviour. The 3DiTALY filament has higher μ in the whole spectral range, with respect to the FILOALFA one. Many could be the causes of the different optical spectra behaviour: the thermal treatment that the filaments have undergone, the presence of some functionalization inside the material (for example, the addition of composites such as TiO2 particles to pure PLA causes a drastic decrease in the transmittance in the UV range, from 80% to 40% [20]), or the dyes employed in the filaments fabrication processes. Unfortunately, the functionalization procedures, the chemical composition of the filament or the dyes employed, are often not clearly indicated in the filament datasheet. To further investigate the cause of the differences in the spectra shown in Fig 2, we performed also XRD measurements on the two samples (data can be found in 10.5281/zenodo.4736126). XRD characterization confirms the amorphous nature of these materials when 3D printed, as already reported by Feng et al. [19]. This analysis also shows the presence of CaCO3 in the 3DiTALY sample, suggesting either that different fabrication processes have been used by the two producers, or the presence of some contamination. Images of the two samples were also acquired with an optical microscope (Inskam 307 LCD Digital Microscope), and different colours were observed. This result suggests us that the different spectra are probably related to the dyes employed by the two producers in the filaments fabrication process.

In the work from Wittbrodt and Pearce, the effects of PLA colour on the material properties of 3D-printed components are shown [21]. It was already known that the colour of the filament can introduce differences in the strength of the printed material and that the polymers contain different degrees of crystallinity depending on the processing history and temperature. They clearly showed also that the percent crystallinity of a 3D-printed part is colour dependent. In addition, the incorporation of nucleating agents into plastics can modify the crystallization rate (raw PLA has a crystallinity of 0–1%), and their results suggest that some of the colouring agents may be acting as crystallization rate modifiers, and then have influences on the transmittance rate of the material. A reported explanation for the change in crystallinity, and then transparency, is the use of different dyes to colour the PLA material. In the spectra shown in Fig 3, it is clearly visible how the colour of different filaments influences the transmission of light through the sheets. Being these PLA filaments from the same producer and from the same stock, we can guess that they share the same basic composition and manufacturing processes. Furthermore, we have noticed that the higher absorbance values are not always associated to the darker filaments (Figs 3 and 4). This phenomenon could be partly related to light scattering, which is influenced both from the material composition and on the printing geometry of the 3D object.

Furthermore, in the work from Wittbrodt and Pearce, they observed that a printed sample of white PLA filament changed its crystallinity when printed at different extruder’s temperature. They tried with 190°C, 200°C, 210°C and 215°C. To create a link between the crystallinity concept and the light attenuation inside these materials, we did a similar test, obtaining the results shown in Fig 5. We found similar μ (same shape and a vertical translation in particular for smaller wavelengths) for the whole temperatures except for 185°C and 205°C. We verified that for temperature lower than 190°C, the filament was not extruded correctly. For what concern the extrusion temperature at 205°C, we have a completely different behaviour with half of the attenuation at the lowest wavelengths and a flatter behaviour of the spectra. To verify the possible correlation between the spectral behaviour and the crystallinity of the sample, XRD measurements were performed on the thin sheets 3D printed with white MKOEM filament at 205°C and 200°C. No crystallinity peaks were observed in both the samples. On the other hand, we acquired images of the two samples with an optical microscope (Inskam 307 LCD Digital Microscope), showing different structures for the thin sheets: more compact for the filament extruded at 200°C and less compact for the one extruded at 205°C. The different structure of the two samples could affect the scattering coefficient, thus explaining the different spectral behaviour of the extinction coefficient.

ABS is a thermoplastic polymer, which has a higher printing temperature than PLA (210°C-250°C), and it is toxic when heated. It is naturally opaque with a yellow tint when the dispersed phase is based on emulsion particle, and opaque to translucent white for those based on bulk particles. Translucency can be increased through manipulation of the particle size distribution [22]. The absorbance of the pure ABS in the UV-visible range (200–600 nm) is quite low [23, 24], while in the range 1050–1350 nm, it was shown that the reflectance is high (>0.85) [25]. A complete spectra for ABS in the range 200–1000 nm is provided by Neher *et al*. but again for the pure material [26]. As already discuss for PLA, also for ABS we have a lack of information about the spectral characteristics of the printed material. As in the PLA case, we found different μ related to the different filament colours, also if the general trend is a higher μ value, as shown in Fig 6.

To verify the applicability of the previous material for DO applications, we choose the two black PLA filaments, whose spectra are shown in Fig 2. From the trials on the black PVC and on the metallic surface it is already clear that the transmitted light at the detector is not negligible for both NIRS and DCS applications. In Fig 7B, indeed, for FILOALFA filament we observe a not negligible amount of correlated light reaching the detector: this signal is due to the laser light that diffuses though the probe directly reaching the detector. This behaviour is confirmed by the FILOALFA PLA spectrum, which has a lower attenuation with respect to the 3DiTALY one. Also, in the DCS acquisition we can clearly see how, increasing the laser power, there is a linear increase in the detected signal (Fig 8). This phenomenon is enhanced when we substitute the PVC surface with a metallic one, since there is also a contribution to the total counts coming from the reflections with that surface. The impact of having a not completely absorbing probe is not negligible in single photon counting and timing measurements, as showed by the results obtained on the solid phantom with TD-NIRS. In Fig 9 it is clear the presence of an unwanted peak in the signal, which corresponds to the spurious signal registered on the acquisitions with the metallic surface. This peak modifies the reflectance curve’s shape, making impossible the retrieval of the phantom optical properties, as shown in Table 2. Also, in the measurements with the DCS technique on the liquid phantom, we can notice (Fig 10), a higher decay time for the autocorrelation function for the FILOALFA filament, suggesting that the laser light, which is highly coherent, is diffused into the probe and directly reach the detection fiber increasing the coherence of the detected light.

## Conclusion

In this work, we give an optical characterization for 3D printed PLA and ABS, in the form of thin sheets, in the range 400–1300 nm. We also study the possibility to employ these materials in the field of diffuse optics. The optical spectra acquired, and the tests on phantoms, with NIRS and DCS, suggest the need of an optical characterization before their employment for DO techniques, to avoid the use of 3D printed objects transparent at the wavelength of interest. This point is crucial, in particular, in case of 3D printed probes, in particular those ones with free beams travelling inside of them, but also when it is necessary to print 3D parts such as phantom boxes or boxes’ caps and any other kind of supports, where there is an interaction with laser light.
